# Pediatric hypertension prevalence with 1 vs. 2 vs. 3 within-visit blood pressure readings (NHANES 2013-2020)

**DOI:** 10.1038/s41371-026-01188-9

**Published:** 2026-07-29

**Authors:** Sandeep K. RIAR, Donald L. BATISKY, Scott GILLESPIE, Andrew M. SOUTH

**Affiliations:** 1 Department of Pediatrics – Division of Nephrology, St. Louis Children’s Hospital, Washington University School of Medicine, St. Louis, MO USA; 2 Department of Pediatrics, Division of Nephrology & Hypertension Cincinnati Children’s Hospital Medical Center University of Cincinnati College of Medicine, Cincinnati, OH US; 3 Department of Pediatrics, Children’s Healthcare of Atlanta, Emory University School of Medicine, Atlanta, GA 30329 US; 4 Department of Pediatrics-Section of Nephrology, Atrium Health Levine Children’s Brenner Children’s Hospital, Wake Forest University School of Medicine, Winston Salem, US; 5 Division of Public Health Sciences- Department of Epidemiology and Prevention, Wake Forest University School of Medicine, Winston Salem, US; 6 Cardiovascular Sciences Center, Wake Forest University School of Medicine, Winston Salem, NC 27157 US

**Keywords:** Diagnosis, Renovascular hypertension

## Abstract

To compare hypertension prevalence using ‘initial’ blood pressure (BP), ‘subsequent’ BP (averaged second and third readings) and ‘total’ BP (averaged three readings), we included 8–17-year-old youth with three BP readings from NHANES 2013–2020. From 2013–2016, BP was obtained using auscultation (manual protocol, MP) and subsequently, using oscillometry (automated protocol, AP). We excluded subjects with ‘zero’ diastolic blood pressure (DBP). In 5656 subjects, clinical hypertension (high systolic blood pressure [SBP] and/or DBP) prevalence was highest with ‘initial’ BP (350/5 642, 6.2% [95% CI 5.6–6.9%]), lower with ‘subsequent’ BP (289/5 642, 5.1% [4.6–5.7%], *p* < 0.001), and lowest with ‘total’ BP (251/5 642, 4.4% [3.9–5.0%], *p* < 0.001). Both MP and AP groups maintained this trend with a significantly lower hypertension prevalence with ‘total’ vs. ‘subsequent’ BP. An exception to the trend occurred in the AP group for three conditions: diastolic hypertension, hypertension in high BMI and Hispanic subjects. Diastolic hypertension prevalence with ‘subsequent’ DBP (86/2 285, 3.8% [3.1–4.6%]) exceeded that with ‘initial’ DBP (83/2 285, 3.6% [2.9–4.5%]) and was lowest with ‘total’ DBP (59/2 285, 2.6% [2.0–3.3%]). Diastolic hypertension prevalence was higher (126/5 642, 2.2% [1.9 − 2.7%]) with the 2016 European guidelines (same as ‘subsequent’ DBP) than with ‘total’ DBP (94/5 642, 1.7% [1.4 − 2.0%]). Our findings support the inclusion of the first BP reading in BP categorization, as ‘total BP’ leads to lower hypertension classification than ‘subsequent’ BP.

**Figure:**
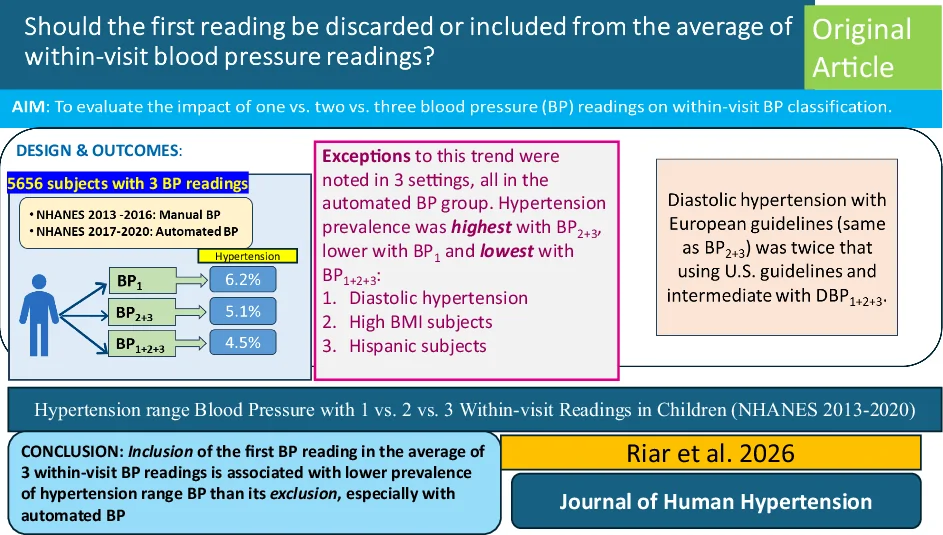

## Introduction

Hypertension guidelines recommend multiple within-visit blood pressure (BP) readings as they lead to lower prevalence of hypertensive BP compared to a single reading [1, 2]. A widespread protocol includes three BP readings, but recommendations differ about the first reading [2, 3]. In the United States (US), adult hypertension guidelines *include* the first reading while pediatric guidelines *exclude* it from the average of within-visit BP readings, similar to European adult and pediatric guidelines [4–7].

The first BP reading is discarded under the assumption that it is the highest, with subsequent readings progressively decreasing. Recent data challenges this supposition [8–10]. The impact of first BP reading on hypertension diagnosis also differs in adults and youth. In adults, hypertension prevalence is lower with the averaged second and third BP readings vs. the average of all three readings (henceforth referred to as ‘subsequent’ and ‘total’ BP respectively) [2, 3]. Pediatric data shows mixed results. Two school-based studies showed results similar to adult data [11, 12]. However, a clinic-based pediatric study showed higher hypertension with ‘subsequent’ than ‘total’ BP but excluded obese children [13]. All three studies used automated BP. Using manual BP readings, a pediatric study compared ‘initial’ vs. ‘total’ BP and concluded that the majority (~ 91%) retained same BP classification, however obese children were more likely to change their BP category [14].

Since these studies, pediatric hypertension guideline updates have lowered hypertension diagnosis thresholds and automated BP use is being favored [5, 15]. In adolescents, the systolic blood pressure (SBP) threshold for hypertension is 11 mmHg higher than normal (≥ 130 vs. < 120 mmHg, hence 130 mmHg is hypertension range while 119 mmHg is normal). Diastolic blood pressure (DBP) threshold is only 1 mmHg higher than normal (≥ 80 vs. <80 mmHg, hence 80 mmHg is hypertension range and 79 mmHg is normal) as DBP is not included in the elevated BP category [4, 5]. However, in younger children, the difference is only about 4 and 3 mmHg respectively (≥95^th^ percentile vs. <90^th^ percentile) [5, 16]. Significant differences in hypertension prevalence based on race and ethnicity have been described in the US in both adults and adolescents [17, 18]. There is limited recent data on how ‘subsequent’, and ‘total’ BP readings affect within-visit BP classification in healthy youth. Importantly, it is unknown if this varies by age, sex, race-ethnicity, obesity or measurement technique (manual vs. automated). Our primary objective was to evaluate the impact of one vs. two vs. three BP readings on BP classification. Our secondary objectives were to determine if the association of number of readings with BP classification varies by (1) age, sex, race-ethnicity and obesity status and; (2) manual vs. automated measurement. In a post-hoc analysis, we compared differences in hypertension classification between pediatric US and European guidelines.

## Patients and methods

### Study design, BP measurement, and classification

The National Health and Nutrition Examination Survey (NHANES) is a series of cross-sectional studies that assess U.S. population’s health and nutrition. Subjects aged ≥8 years have three BP measurements using a standardized protocol during a single visit [19]. We used data from 4 cycles (2013–2014, 2015–2016, 2017–2018 and 2019–2020). Due to the coronavirus disease 2019 pandemic, data for the 2019–2020 cycle was incomplete and combined with the 2017–2018 cycle data to form a nationally representative sample [20]. We included 8-–17-year-old subjects with three BP readings. We excluded subjects with ‘zero’ DBP, as averaging these values would skew the data [21]. NHANES data collection is approved by the National Center for Health Statistics Research Ethics Review Board (protocol #2011 − 17 for years 2011 − 2016, #2011 − 17 followed by #2018 − 01 for the year 2017 and #2018-01 for years 2018 − 2020). Parental informed consent and participant assent were obtained for 7–17-year-old subjects [22].

Demographic information (age, sex, race-ethnicity) was collected during an interview [23]. The race-ethnicity variable, derived from self-report about Hispanic ethnicity and race, was classified into four categories: non-Hispanic White, non-Hispanic Black, Hispanics, and miscellaneous. Standing height and weight were measured using standard protocol [24]. Body mass index (BMI) percentile by age and sex was determined from the Centers for Disease Control and Prevention 2000 growth charts [25]. Weight status was categorized dichotomously as underweight/healthy weight (BMI <85th percentile) vs. overweight/obesity (BMI ≥85th percentile).

BP methodology changed during the study period. During the 2013–2016 cycles, auscultatory BP was measured with a mercury sphygmomanometer, referred to as the manual protocol (MP) [26]. MP included three BP readings thirty seconds apart. In the 2017–2018 cycle, NHANES added oscillometric BP measurement using an automated protocol (AP) and BP was measured using both MP and AP [23, 27]. Since 2019 only AP has been used [19]. The AP utilized the Omron HEM-907XL device (Omron Healthcare, Kyoto, Japan) that automatically obtained three BP measurements one minute apart [19, 23, 27]. For the present analysis, the MP group included manual readings from 2013–2016, and the AP group included automated readings from the 2017–March 2020 pre-pandemic public-use file. Public-use NHANES files did not permit subject-level linkage of manual and automated triplicate BP measurements for the same 2017–2018 participants; therefore, a within-subject paired analysis was not possible. We compared findings between the MP and AP groups, although they included different subjects because 1) NHANES uses a standardized, rigorously stratified sampling method across all cycles; (2) we used unweighted data because our objective was to obtain standardized BP data in a large pediatric sample rather than estimating US population parameters [20, 28].

BP categories of ‘normal BP’, ‘elevated BP’ (EBP) and ‘hypertension’ were defined using the 2017 American Academy of Pediatrics hypertension guidelines [5]. As hypertension diagnosis requires three visits with high BP, and NHANES is limited to a single encounter, hypertension disorder classification here refers to ‘single visit’ BP classification. Specifically, for 8–12-year-old subjects, we determined the BP percentile using age, sex, and height. Normal BP referred to SBP and DBP <90^th^ percentile for 8–12-year-old subjects and <120/ < 80 mm Hg for 13–17-year-old subjects. In 8–12-year-old subjects, EBP referred to SBP and/or DBP ≥ 90^th^ percentile to <95^th^ percentile or 120/80 mm Hg to <95^th^ percentile (whichever was lower). EBP in 13–17-year-old subjects was defined as SBP 120- < 130 mm Hg with DBP < 80 mm Hg. Hypertension was defined in 8–12-year-old subjects as SBP and/or DBP ≥95^th^ percentile or ≥130/ ≥ 80 mm Hg (whichever was lower). For 13–17-year-old subjects, hypertension was defined by BP ≥ 130/ ≥ 80 mm Hg.

‘Initial’ BP refers to the first BP reading, ‘subsequent’ BP refers to the averaged second and third readings and ‘total’ BP indicates the average of all three readings. US guidelines specify a single BP reading; if this is high, two additional BP readings are obtained and averaged for BP classification [5]. European guidelines recommend three within-visit BP readings and use the average of second and third BP reading (same as ‘subsequent’ BP) [7].

### Statistical analysis

Analyses were conducted on NHANES 2013–2020 as a convenience sample of available

BP measurements; complex survey weights and design variables were not applied. Results are intended for methodological comparisons rather than population-level inference. Demographics and BP readings were summarized using medians (IQR) for continuous variables and frequencies (%) for categorical data. Concordance between initial, subsequent, and total BP readings was assessed with McNemar’s tests. BP measures were compared between manual and automatic readings using Wilcoxon rank-sum tests and Chi-square or Fisher’s exact tests. Analyses were performed in SAS v.9.4 (Cary, NC) with a significance threshold of 0.05. One author (SR) had full access to the data and ensured its integrity.

## Results

**Fig. 1:**
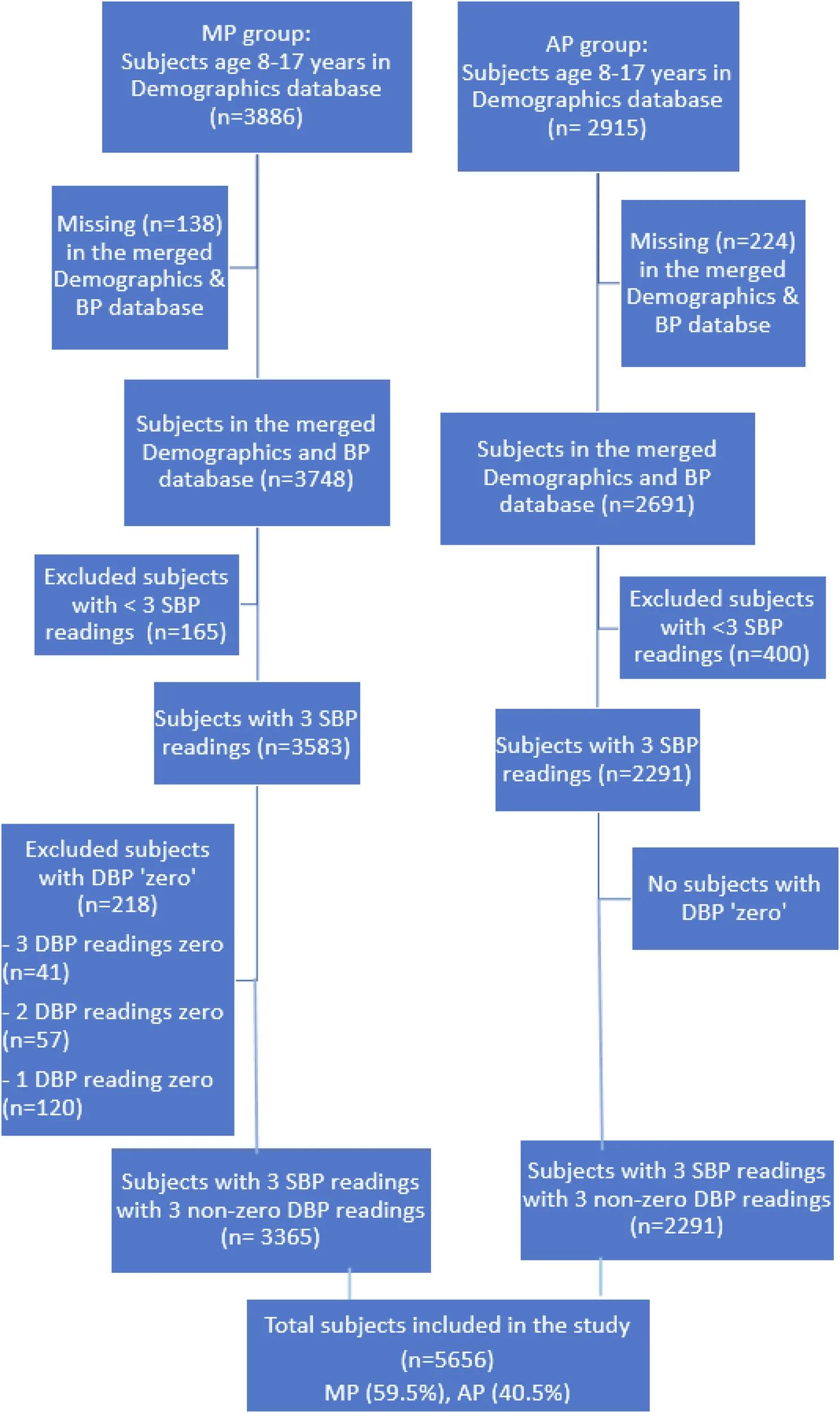
CONSORT flow diagram demonstrating selection of eligible subjects included in the primary analysis. [Reprinted from “Within-visit blood pressure variability in children and adolescents in the National Health and Nutrition Examination Survey (2013–2020),” by Riar SK, Gillespie S, South AM, J Hypertens. 2025 Jul 1;43(7):1158–1168. Figure 1. Copyright 2025 by Wolters Kluwer Health, Inc. Used with permission].

Of the 6 801 eligible subjects, 5 874 (86.4%) had three BP readings (Fig. 1). After excluding 218 subjects with ‘zero’ DBP, we included 5 656 subjects with 3 365 (59.5%) in the MP and 2 291 (40.5%) in the AP group.

**Fig. 2:**
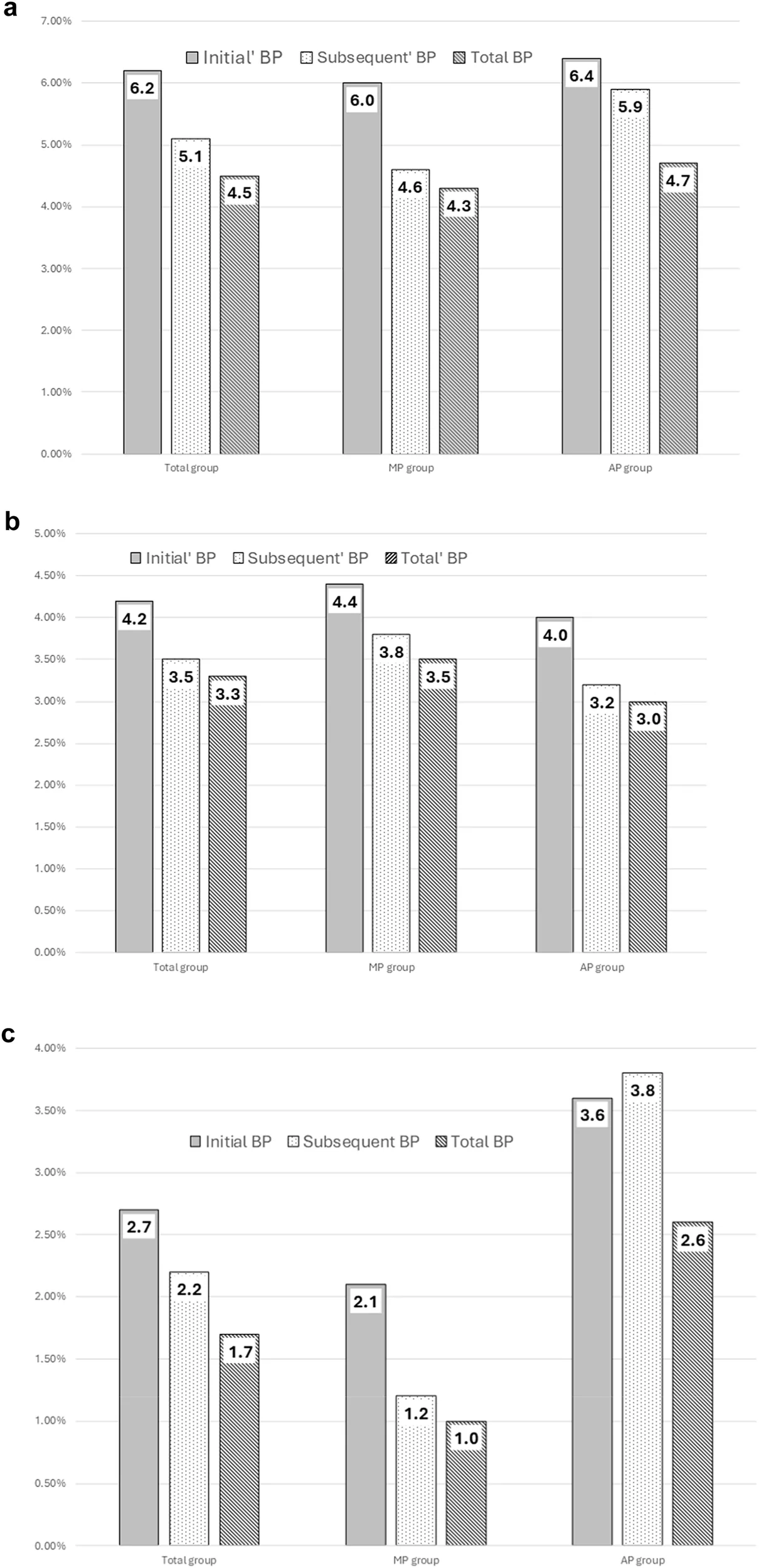
Impact of ‘initial’, ‘subsequent’ and ‘total’ BP on hypertension prevalence. **a** Hypertension range SBP and/or DBP with ‘initial’, ‘subsequent’ and ‘total’ BP. **b** Hypertension range SBP with ‘initial’, ‘subsequent’ and ‘total’ BP. **c** Hypertension range DBP with ‘initial’, ‘subsequent’ and ‘total’ BP.

Table 1 shows the basic characteristics of subjects. MP and AP groups had similar age, sex, and BMI distributions; however, MP group had more Hispanic subjects. ‘Clinical’ hypertension (i.e. hypertension-range SBP and/or DBP) prevalence was similar in MP and AP groups with ‘initial’ and ‘total’ BP, however it was higher in the AP group with ‘subsequent’ BP (Fig. 2a, supplementary Table S1). For clarity, we present our findings by first comparing hypertension prevalence using ‘initial’, ‘subsequent’, and ‘total’ BP, followed by comparing MP and AP groups. Supplementary Table S5 summarizes descriptive patterns of the three within-visit BP readings, including which reading was highest and the frequency of consistently increasing or decreasing patterns.

**Table 1:** Characteristics of study subjects.

	Overall *N* = 5656	MP group *N* = 3365	AP group *N* = 2291	*P*-value^2,3^
Median Age [IQR], years	12.0 (10.0, 15.0)	12.0 (10.0, 15.0)	12.0 (10.0, 15.0)	0.83
Age: 8–12 years	2996 (53%)	1778 (52.8%)	1218 (53.2%)	0.81
Age: 13–17 years	2660 (47%)	1587 (47.2%)	1073 (46.8%)	
Sex				0.83
Male	2829 (50%)	1687 (50.1%)	1142 (49.9%)	
Female	2827 (50%)	1678 (49.9%)	1149 (50.2%)	
BMI Category^1^				0.08
Underweight/Normal	3344 (59.4%)	2021 (60.4%)	1323 (58%)	
Overweight/Obesity	2282 (40.6%)	1325 (39.6%)	957 (42%)	
Race-ethnicity				<0.001
Non-Hispanic White	1650 (29.2%)	945 (28.1%)	705 (30.8%)	
Non-Hispanic Black	1377 (24.4%)	770 (22.9%)	607 (26.5%)	
Hispanic	1697 (30%)	1130 (33.6%)	567 (24.8%)	
Miscellaneous	932 (16.5%)	520 (15.5%)	412 (18%)	

Median (IQR) or N (column %). Bold font indicates statistically significant results at p < 0.05.

^1^N = 30 subjects had missing weight and/or height measurements; hence BMI could not be determined.

^2^P-values here compare MP and AP groups and are calculated by Wilcoxon sum-rank tests for continuous data and Fisher’s exact and/or Chi-squared tests for categorical data.

^3^Being an exploratory analysis, all p-values are presented unadjusted for multiple comparisons.

In comparing ‘initial’, ‘subsequent’ and ‘total’ BP, we observed a consistent trend: clinical hypertension prevalence was highest with ‘initial’ BP (350/5 642, 6.2% [95% CI 5.6–6.9%]), lower with ‘subsequent’ BP (289/5 642, 5.1% [4.6–5.7%]), and lowest with ‘total’ BP (251/5 642, 4.4% [3.9–5.0%]). This pattern held for ‘clinical’ hypertension (Fig. 2a) and ‘systolic’ hypertension (Fig 2b) in both MP and AP groups. Notably, ‘clinical’ hypertension prevalence with ‘total’ BP was significantly lower than with ‘subsequent’ BP in both MP (4.3 vs. 4.6%, *p* = 0.048) and AP group (4.7 vs 5.9%, *p* < 0.001) (Supplementary Table S1). Systolic hypertension prevalence was similar in the MP and AP groups, whereas diastolic hypertension prevalence was significantly higher in the AP group than in the MP group. ‘Diastolic’ hypertension showed the same trend in MP but *not* in AP group (Fig 2c). Specifically, ‘diastolic’ hypertension prevalence in the AP group with ‘subsequent’ DBP (3.8% [3.1–4.6%]) exceeded that with ‘initial’ DBP (3.6% [2.9–4.5%]) and was lowest with ‘total’ DBP (2.6% [2.0–3.3%]). While the difference between ‘diastolic’ hypertension with ‘subsequent’ and ‘initial’ DBP was not significant, hypertension prevalence with ‘total’ DBP was significantly lower than with ‘subsequent’ DBP (2.6 vs. 3.8%, *p* < 0.001) (Supplementary Table S1).

Regardless of age, sex, BMI and race-ethnicity, ‘clinical’ hypertension exhibited the same general trend, with two exceptions, both in the AP group (Supplementary Table S2) with hypertension prevalence being highest with ‘subsequent’ and not ‘initial’ BP. In AP group Hispanic subjects, ‘clinical’ hypertension prevalence was 4.9% [3.4−7.0%], 4.8% [3.3−6.8%] and 3.2% [2.0−5.0%] with ‘subsequent’ BP, ‘initial’ and ‘total’ BP respectively (Supplementary Figure S1b). Similarly, in high BMI subjects, the corresponding prevalence was 7.1% [5.8−8.6%], 6.8% [5.9−7.9%] and 6.5% [5.1−8.2%] (Supplementary Figure S1a). In both subgroups, ‘clinical’ hypertension prevalence with ‘total’ BP was significantly lower than with ‘initial’ BP. Remaining demographic groups demonstrated the typical trend. Although ‘clinical’ hypertension prevalence was lower with ‘subsequent’ than with ‘initial’ BP, statistical significance varied (Supplementary Table S2). In contrast, ‘clinical’ hypertension prevalence was *significantly* lower with ‘total’ vs. ‘initial’ BP in nearly all demographic groups.

In a sub-group analysis, we used ‘initial’ BP to categorize all subjects into three subgroups– ‘hypertension range BP’, ‘EBP range BP’ and ‘normal’–separately for SBP and DBP (Supplementary Tables S3a-c, Figure S2a). In the first subgroup–subjects with hypertension range ‘initial’ DBP – fewer than half retained the diastolic hypertension category with ‘subsequent’ and ‘total’ DBP (37.9 and 46.4% respectively) (Supplementary Table S3a, Figure S2b). In the second sub-group–subjects with EBP-range ‘initial’ BP–only 66 subjects had EBP-range DBP; therefore, we focused on SBP. Reclassification from EBP to hypertension was more common with ‘subsequent’ vs. ‘total’ SBP for both the MP group (6.1 vs. 3.3%, *p* = 0.003) and the AP group (11.1 vs. 8.2%, *p* = 0.025) (Supplementary Table S3b, Figure S2c). In the third subgroup–those with normal ‘initial’ BP–reclassification to a higher BP category (EBP/hypertension) was more common with ‘subsequent’ vs. ‘total’ BP (Supplementary Table S3c). For SBP, reclassification from normal to EBP/hypertension with ‘subsequent’ vs. ‘total’ SBP was 2.2 vs. 0.8% in the MP group (*p* < 0.001) and 3.8 vs. 2.2% in the AP group (*p* < 0.001). For DBP, this was 1.0 vs 0.4% in the MP group (*p* < 0.001) and 2.7 vs. 1.2% in the AP group (*p* < 0.001).

Finally, when applying European and US hypertension guidelines to our subjects, prevalence of hypertension-range SBP was similar under both approaches and similar to ‘total’ SBP (Table 2). In contrast, diastolic hypertension prevalence was highest with the 2016 European guidelines (same as ‘subsequent’ DBP), lower with ‘total’ DBP, and lowest with the 2017 United States guidelines (2.2, 1.7, and 1.1%, respectively).

**Table 2:** Prevalence of hypertension-range SBP and DBP with ‘initial’ BP, ‘subsequent’ BP, ‘total’ BP, U.S. guidelines, and European guidelines.

	‘Initial’ BP	^2^’Subsequent’ BP (Average of 2^nd^ and 3^rd^ BP readings)	‘Total’ BP (Average of all three BP readings)	U.S. Guidelines (If initial BP reading high, 2 additional BP readings obtained & averaged)	^2^European Guidelines (Average of 2^nd^ and 3^rd^ BP readings)
^1^Hypertension range SBP	237/5642 4.2% [3.7%–4.8%]	199/5642 3.5% [3.1%–4.0%]	187/5642 3.3% [2.9%–3.8%]	185/5642 3.3% [2.8%–3.8%]	199/5642 3.5% [3.1%–4.0%]
^1^Hypertension range DBP	153/5642 2.7% [2.3%–3.2%]	126/5642 2.2% [1.9%–2.7%]	94/5642 1.7% [1.4%–2.0%]	64/5642 1.1% [0.9%–1.4%]	126/5642 2.2% [1.9%–2.7%]

^1^N = 14 subjects age<13 years had no height measurements and BP percentiles could not be calculated.

^2^European Guidelines’ hypertension BP protocol is the same as ‘subsequent’ BP in our study.

## Discussion

Our analysis of NHANES data collected in community dwelling youth 8–17 years of age who underwent triplicate BP measurements at one visit showed that ‘clinical’ hypertension prevalence was highest with ‘initial’ BP, lower with ‘subsequent’ BP and lowest with ‘total’ BP, in both MP and AP groups. We also observed three exceptions to this trend in the AP group whereby prevalence of hypertension range BP with ‘subsequent’ BP *exceeded* that with ‘initial’ BP and still was *lowest* with ‘total’ BP. These included diastolic hypertension, ‘clinical’ hypertension in Hispanic subjects and those with high BMI.

Our finding that ‘clinical’ hypertension prevalence was highest with ‘initial’ BP, lower with ‘subsequent’ and lowest with ‘total’ BP (6.2, 5.1 and 4.5% respectively) contrasts with adult trends. Hypertension prevalence using ‘initial’, ‘subsequent’ and ‘total’ BP was 22.9, 18.6 and 19.3% respectively in US adults with manual BP and 31.7, 24.0 and 25.3% respectively in Taiwanese adults with automated BP [2, 3]. Two school-based pediatric studies echoed adult trends. In Chinese schoolchildren, hypertension prevalence was 23.4, 17.2 and 17.8% with ‘initial’, ‘subsequent’ and ‘total’ BP, respectively [11]. In Swiss schoolchildren, the corresponding prevalence was 21.2, 11.6 and 13.6% [12]. In contrast, our findings align with a clinic-based pediatric Italian study with corresponding hypertension prevalence of 13, 8.9 and 6.8%, respectively [13]. Both school-based studies had substantially higher hypertension prevalence than our study and the Italian study raising concerns about clinical applicability of school-based hypertension studies. The prevalence of hypertensive BP in our study is closer to the estimated 2─5% range for pediatric hypertension [29]. Our finding of lower hypertension prevalence with ‘total’ vs. ‘subsequent’ BP, challenges the assumption that ‘initial’ BP is *always* the highest and BP readings decline progressively across repeated measurements–an observation largely supported by data reporting group-level BP readings [12, 30]. Recent studies analyzing individual-level BP trends, including our study, demonstrate that first BP reading is *usually* but *not always* the highest and successive readings typically exhibit a fluctuating rather than consistently decreasing pattern [8–10, 31]. The probable explanation for prevalence of hypertensive BP being highest with ‘initial’ BP is that the first BP is often the highest of three readings, attributed to an ‘alerting reaction’ to BP measurement–a physiologic stress, similar to a *fight-or-flight* response [32]. In our study, highest SBP was the first, second and third SBP in 44, 31 and 25% subjects respectively [31]. Additionally, ‘initial’ BP represents a single reading, leading to random variability and low reliability, considering BP variability (BPV) [4]. The difference in hypertensive BP prevalence between ‘subsequent’ vs. ‘total’ BP may be influenced by: (1) the proportion of individuals with ‘initial’ BP as the highest; (2) BPV. In the Taiwanese adult study, 54% of subjects had their highest reading with ‘initial’ BP, while in our cohort, the majority (56%) had the second or third reading as the highest. This may explain our higher hypertension prevalence with ‘subsequent’ than ‘total’ BP. BPV and phenomena such as ‘zero’ DBP–rare in adults–are more common in youth [10, 21]. Hence, youth may be more likely to have an ‘initial’ BP lower than successive readings explaining lower hypertension prevalence with ‘total’ vs. ‘subsequent’ BP. Higher pediatric BPV also suggests better reliability with three vs. two BP readings [33].

An exception to the aforementioned trend in the prevalence of hypertensive BP was noted in the AP group for diastolic hypertension (highest with ‘subsequent’, and lowest with ‘total’ BP). The higher occurrence of hypertensive DBP with subsequent’ vs. ‘initial’ ‘DBP in the AP group–but not MP group–appears to be due to group-level DBP in AP group being about 5 mmHg higher than group-level DBP in MP group (Supplementary Table S4). This finding combined with over half the subjects whose highest DBP was the second/third reading (AP: 56%, MP: 59%) likely allowed ‘subsequent’ DBP to exceed hypertension threshold more often than ‘initial’ BP. Other studies have noted higher DBP with automated vs. manual technique [34]. One possible explanation is the use of adult-level ‘deflation threshold’ of 40–45 mm Hg in automated devices [34]. Deflation threshold, the pressure at which remaining air is released from the valve during deflation, is typically set below the expected adult DBP. While appropriate for adults, a deflation threshold of 40–45 mmHg in youth risks missing the actual DBP, which tends to be lower. In our study, the lower prevalence of diastolic hypertension in MP than AP group (Supplementary Table S1) may be related to DBP values as low as 2–14 mmHg despite excluding subjects with ‘zero’ DBP [31].

Our subsequent analyses yielded three notable findings: First, although subgroup analysis of subjects with hypertensive ‘initial’ BP showed a higher persistence of hypertension classification with ‘total’ vs. ‘subsequent’ BP, in the overall cohort, hypertensive BP prevalence was lower with ‘total’ vs. ‘subsequent’ BP. Second, among subjects with ‘initial’ BP in the ‘normal/EBP’ range, hypertensive BP prevalence was nearly twice as high with ‘subsequent’ vs. ‘total’ BP (Supplementary Table S3d). Third, prevalence of hypertensive DBP with European guidelines was twice that with U.S. guidelines (2.2 vs. 1.1%), which raises the possibility that European guidelines, by consistently excluding the first BP, may lead to overdiagnosis of diastolic hypertension. Conversely, U.S. guidelines may underdiagnose it while ‘total’ DBP leads to a rate intermediate between the two guidelines. All three findings may be related to BPV, and specifically the proportion of subjects with sufficiently lower first BP reading–so that its inclusion in ‘total’ BP led to lower pediatric hypertension prevalence.

The difference in the highest and lowest clinical hypertension prevalence among the three BP approaches in this study was 1.7% in both MP and AP groups (Fig. 2a, Table S1), which may appear small. However, we feel this is clinically relevant for three reasons: 1) pediatric hypertension prevalence is estimated at 2–5% so a 1.7% difference represents 34–85% change; 2) with universal BP measurement at annual physical examinations, 1.7% prevalence difference at the population level can lead to BP misclassification in thousands of patients every year; 3) the NHANES BP protocol is highly standardized and the difference in real-life clinical settings may be higher [29]. Nonetheless, additional studies would be needed to investigate the effect of this variability on ≥3 measurements on different days per guidelines [5].

The role of the first BP reading in classifying pediatric hypertension warrants further discussion. Several factors support its inclusion in within-visit BP classification. First, emerging data including ours confirms that first BP reading isn’t always the highest and successive readings may be higher [2, 10, 31]. Second, with rising trend in obesity and use of automated BP, ‘subsequent’ BP may yield higher hypertension prevalence than ‘initial’ BP, while ‘total’ BP leads to consistently lower hypertension prevalence. Third, our findings suggest that European guidelines, which exclude the first BP reading, may over-diagnose diastolic hypertension. Fourth, the normative data used to establish BP percentiles in pediatric guidelines was derived from the first BP reading [35].

Our study has limitations. First, it is cross-sectional and based on a single visit, limiting inference about hypertension diagnosis which requires three visits. Nonetheless, the BP protocol used at a single visit matters as it leads to follow-visits to confirm BP abnormality and potentially higher rate of referrals. Second, the NHANES BP protocol is standardized, limiting its generalizability to routine clinical settings. Third, we lacked data on target organ injury. Fourth, we lacked information about antihypertensive medication use. Lastly, MP and AP groups consisted of different subjects. However, each NHANES cycle uses a rigorously consistent sampling methodology, and the large sample size in both groups provided a reasonable basis for comparison [20, 28]. The ideal BP protocol for diagnosing hypertension should include target organ injury, however the unavailability of echocardiogram data in the NHANES database, as an example of target organ injury, makes it difficult. Some researchers recommend the diagnostic strategy resulting in the lowest hypertension prevalence as a proxy for accuracy [2]. By that criterion, ‘total’ BP–which produced the lowest prevalence of hypertensive BP in our study–may be considered the most optimal among the three approaches. The strengths of our study include a large sample size and inclusion of both manual and automated BP technique.

## Conclusions

In a large, community-based sample of youth, prevalence of ‘clinical’ hypertension (hypertension range SBP and/or DBP) was highest with ‘initial’ BP, lower with ‘subsequent’ BP and lowest with ‘total’ BP, irrespective of manual or automated BP measurements. Exceptions to this trend were noted in the AP group, including diastolic hypertension, ‘clinical’ hypertension in Hispanic subjects and in those with high BMI. In these subgroups, prevalence of hypertensive BP was higher with ‘subsequent’ vs. ‘initial’ BP, and still lowest with ‘total’ BP. Our study also suggests that diastolic hypertension may be over-diagnosed with the 2016 European guidelines, underdiagnosed by the 2017 US guidelines and intermediate with ‘total’ BP. Importantly, our findings support the inclusion of the first BP reading in BP categorization, as it leads to consistently lower prevalence of hypertensive BP as compared to its exclusion from the average of three ‘within-visit’ BP readings.

## Summary

### What is known about topic?

- The first BP reading is discarded from the average of within-visit BP readings by European adult and pediatric hypertension guidelines and United States (US) pediatric hypertension guidelines but included by US adult hypertension guidelines.
- This is based on an assumption that the first of multiple within-visit BP readings is the highest and successive readings progressively decrease.

### What this study adds?

- Clinical hypertension prevalence was highest with ‘initial BP’, lower with the ‘average of the 2^nd^ and 3^rd^ BP readings’ and lowest with the ‘average of all three BP readings’. This trend held true for both manual and automated BP groups.
- Exceptions to this trend were noted in the automated BP group for high BMI and Hispanic subjects. Hypertension prevalence with the ‘averaged 2^nd^ and 3^rd^ readings’ surpassed that with ‘initial BP reading’ and was still lowest with the ‘averaged 3 BP readings’.
- In children, hypertension prevalence was *higher* when the first BP reading was *excluded* from the average of three within-visit BP readings than when it was included.

## Supplementary information

Supplemental material

## Data Availability

All data analyzed in this manuscript are publicly available from the NHANES datasets via the CDC (Centers for Disease Control and Prevention) website: https://www.cdc.gov/nchs/nhanes/index.html. Blood pressure percentiles were calculated directly from NHANES data using the guidance from Flynn et al. (https://publications.aap.org/pediatrics/article/140/3/e20171904/38358/Clinical-Practice-Guideline-for-Screening-and).
