# Supplementary material for: Pediatric hypertension prevalence with 1 vs. 2 vs. 3 within-visit blood pressure readings (NHANES 2013-2020)
Source: J Hum Hypertens. 2026 Jul 29;40(9):739–46. doi: 10.1038/s41371-026-01188-9 (PMC13558048; doi:10.1038/s41371-026-01188-9)
Supplement: Supplementary file 1 [file 41371_2026_1188_MOESM1_ESM.doc]

**ONLINE RESOURCES (SUPPLEMENTARY DATA) for**

**Article title:** **Hypertension range Blood Pressure with 1 vs. 2 vs. 3 Within-visit Readings in Children (NHANES 2013-2020)**

**Journal name**: Journal of Human Hypertension

**Author names**: SK Riar^a^, MD, DL Batisky^b^, MD, Scott Gillespie^c^, MS, MPSH, Andrew M. South^d^, MD, MS

**Affiliations:** ^a^St. Louis Children’s Hospital, Washington University School of Medicine, St. Louis, Missouri; ^b^Cincinnati Children’s Hospital Medical Center, University of Cincinnati College of Medicine, Cincinnati, Ohio; ^c^Children’s Healthcare of Atlanta, Emory University School of Medicine, Atlanta, Georgia; ^d^Atrium Health Levine Children’s Brenner Children’s Hospital, Wake Forest University School of Medicine, Winston Salem, North Carolina.

**Corresponding author:**

- SK Riar: Email: [sandeepriar.md@gmail.com](mailto:sandeepriar.md@gmail.com)

**Figure legends:**

**Figure S1a: ‘Clinical’ hypertension stratified by age, sex, and BMI (MP vs. AP groups)**

**Figure S1b: ‘Clinical’ hypertension stratified by race-ethnicity (MP vs. AP groups)**

**Figure S2a: Subjects with ‘initial’ SBP in hypertension range: impact of ‘subsequent’ vs. ‘total’ BP on BP classification**. [Each column includes subjects with ‘initial’ SBP in hypertension range in MP or AP group as indicated. Impact of ‘subsequent’ SBP on BP category (normal, EBP or hypertension) is shown in the first two columns. The next two columns show change in BP category with ‘total’ SBP.]

**Figure S2b: Subjects with ‘initial’ DBP in hypertension range: impact of ‘subsequent’ vs. ‘total’ BP on BP classification.** [Each column includes subjects with ‘initial’ DBP in hypertension range in MP or AP group as indicated. Impact of ‘subsequent’ DBP on BP category (normal, EBP or hypertension) is shown in the first two columns. The next two columns show change in BP category with ‘total’ DBP.]

**Figure S2c: Subjects with ‘initial’ SBP in EBP range: impact of ‘subsequent’ vs. ‘total’ BP on BP classification** [Each column includes subjects with ‘initial’ SBP in EBP range in MP or AP group as indicated. Impact of ‘subsequent’ SBP on BP category (normal, EBP or hypertension) is shown in the first two columns. The next two columns show change in BP category with ‘total’ SBP.]

**Table legends:**

**Table S1: Hypertension range BP with ‘initial’, ‘subsequent’ and ‘total’ BP. [**Bold font indicates statistically significant results at p <0.05.

95% confidence intervals were calculated using the Wilson method for binomial proportions.

^1^N = 14 subjects aged <13 years had no height measurements and BP percentiles could not be calculated.

^2^Clinical hypertension refers to the presence of SBP and/or DBP in the hypertension range.

^3^P-values compare MP and AP groups and were calculated using chi-squared or Fisher’s exact tests, as appropriate.

^4^P-values for paired within-group comparisons were calculated using McNemar’s tests.

^5^Being an exploratory analysis, all p-values are presented unadjusted for multiple comparisons.]

**Table S2: ‘Clinical’ hypertension with ‘initial’, ‘subsequent’ and ‘total’ BP by demographic characteristics [**Bold font indicates statistically significant results at p <0.05.

95% confidence intervals were calculated using the Wilson method for binomial proportions.

^1^Clinical hypertension refers to SBP and/or DBP in the hypertension range.

^2^N = 14 subjects aged <13 years had no height measurements and BP percentiles could not be calculated.

^3^N = 30 subjects had missing weight and/or height measurements; therefore, BMI could not be determined. ^4^P-values were calculated using McNemar’s tests and are unadjusted for multiple comparisons.]

**Table S3a: Subjects with ‘initial’ BP in hypertension range: impact of repeat BP readings on BP category. [**^1^N = 14 subjects aged <13 years had no height measurements and BP percentiles could not be calculated.

^2^P-values compare MP and AP groups and were calculated using chi-squared or Fisher’s exact tests, as appropriate.

^3^SBP1/DBP1 = initial SBP/DBP reading; SBP2+3/DBP2+3 = subsequent SBP/DBP; SBP1+2+3/DBP1+2+3 = total SBP/DBP.]

**Table S3b**: **Subjects with ‘initial’ BP in ‘elevated BP’ range: impact of repeat BP readings on BP category. [**^1^N = 14 subjects aged <13 years had no height measurements and BP percentiles could not be calculated.

^2^P-values compare MP and AP groups and were calculated using chi-squared or Fisher’s exact tests, as appropriate. ^3^SBP1/DBP1 = initial SBP/DBP reading; SBP2+3/DBP2+3 = subsequent SBP/DBP; SBP1+2+3/DBP1+2+3 = total SBP/DBP.]

**Table S3c**: **Subjects with ‘initial’ BP in normal range: impact of repeat BP readings on BP category [**^1^N = 14 subjects aged <13 years had no height measurements and BP percentiles could not be calculated.

^2^P-values compare MP and AP groups and were calculated using chi-squared or Fisher’s exact tests, as appropriate. ^3^SBP1/DBP1 = initial SBP/DBP reading; SBP2+3/DBP2+3 = subsequent SBP/DBP; SBP1+2+3/DBP1+2+3 = total SBP/DBP.]

**Table S3d:** **Subjects with ‘initial’ BP in ‘EBP’ or normal range: impact of repeat BP readings on BP category. [**^1^N = 14 subjects aged <13 years had no height measurements and BP percentiles could not be calculated. ^2^P-values compare MP and AP groups and were calculated using chi-squared or Fisher’s exact tests, as appropriate. ^3^SBP1/DBP1 = initial SBP/DBP reading; SBP2+3/DBP2+3 = subsequent SBP/DBP; SBP1+2+3/DBP1+2+3 = total SBP/DBP.]

**Table S4: Group level BP readings. [**^1^P-values compare MP and AP groups and were calculated using Wilcoxon rank-sum tests.]

**Table S5: Descriptive patterns of three within-visit systolic and diastolic BP readings in the manual and automated groups.** [Reprinted from “Within-visit blood pressure variability in children and adolescents in the National Health and Nutrition Examination Survey (2013-2020),” by Riar SK, Gillespie S, South AM, J Hypertens. 2025 Jul 1;43(7):1158-1168. Table 3. Copyright 2025 by Wolters Kluwer Health, Inc. Used with permission.]

**Table S1:** **Hypertension range BP with ‘initial’, ‘subsequent’ and ‘total’ BP**

|  | **Overall**  **N=5,656**  **n/N, % [95% CI]** | **MP group**  **N=3,365**  **n/N, % [95% CI]** | **AP group**  **N=2,291**  **n/N, % [95% CI]** | **P-value^3,5^** |
| --- | --- | --- | --- | --- |
| **Clinical Hypertension range BP^1,2^** |  |  |  |  |
| Initial SBP and/or DBP | 350/5642  6.2% [5.6%–6.9%] | 203/3357  6.0% [5.3%–6.9%] | 147/2285  6.4% [5.5%–7.5%] | 0.56 |
| Subsequent SBP and/or DBP | 289/5642  5.1% [4.6%–5.7%] | 154/3357  4.6% [3.9%–5.3%] | 135/2285  5.9% [5.0%–7.0%] | **0.03** |
| Total SBP and/or DBP | 251/5642  4.4% [3.9%–5.0%] | 143/3357  4.3% [3.6%–5.0%] | 108/2285  4.7% [3.9%–5.7%] | 0.40 |
| **P-Value^4,5^** |  |  |  |  |
| Initial vs. Subsequent SBP and/or DBP | **<0.001** | **<0.001** | 0.300 |  |
| Initial vs. Total SBP and/or DBP | **<0.001** | **<0.001** | **<0.001** |  |
| Subsequent vs. Total SBP and/or DBP | **<0.001** | **0.048** | **<0.001** |  |
| **Hypertension range SBP^1^** |  |  |  |  |
| Initial SBP | 237/5642  4.2% [3.7%–4.8%] | 146/3357  4.3% [3.7%–5.1%] | 91/2285  4.0% [3.3%−4.9%] | 0.5 |
| Subsequent SBP | 199/5642  3.5% [3.1%–4.0%] | 126/3357  3.8% [3.2%−4.5%] | 73/2285  3.2% [2.5%–4.0%] | 0.26 |
| Total SBP | 187/5642  3.3% [2.9%–3.8%] | 118/3357  3.5% [2.9%–4.2%] | 69/2285  3.0% [2.4%–3.8%] | 0.31 |
| **P-Value^3,4^** |  |  |  |  |
| Initial vs. Subsequent SBP | **0.002** | **0.015** | **0.039** |  |
| Initial vs. Total SBP | **<0.001** | **<0.001** | **0.003** |  |
| Subsequent vs. Total SBP | 0.058 | 0.059 | 0.394 |  |
| **Hypertension range DBP^1^** |  |  |  |  |
| Initial DBP | 153/5642  2.7% [2.3%–3.2%] | 70/3357  2.1% [1.7%–2.6%] | 83/2285  3.6% [2.9%−4.5%] | **<0.001** |
| Subsequent DBP | 126/5642  2.2% [1.9%−2.7%] | 40/3357  1.2% [0.9%–1.6%] | 86/2285  3.8% [3.1%–4.6%] | **<0.001** |
| Total DBP | 94/5642  1.7% [1.4%–2.0%] | 35/3357  1.0% [0.8%–1.4%] | 59/2285  2.6% [2.0%–3.3%] | **<0.001** |
| **P-Value^4,5^** |  |  |  |  |
| Initial vs. Subsequent DBP | **0.034** | **<0.001** | 0.761 |  |
| Initial vs. Total DBP | **<0.001** | **<0.001** | **0.001** |  |
| Subsequent vs. Total DBP | **<0.001** | 0.197 | **<0.001** |  |

Bold font indicates statistically significant results at p <0.05.

95% confidence intervals were calculated using the Wilson method for binomial proportions.

^1^N = 14 subjects aged <13 years had no height measurements and BP percentiles could not be calculated.

^2^Clinical hypertension refers to the presence of SBP and/or DBP in the hypertension range.

^3^P-values compare MP and AP groups and were calculated using chi-squared or Fisher’s exact tests, as appropriate.

^4^P-values for paired within-group comparisons were calculated using McNemar’s tests.

^5^Being an exploratory analysis, all p-values are presented unadjusted for multiple comparisons.

**^Table S2: ‘Clinical’ hypertension with ‘initial’, ‘subsequent’ and ‘total’ BP by demographic characteristics^**

|  | **Clinical Hypertension with ‘initial’ BP^1^**  **n/N, % [95% CI]** | **Clinical Hypertension with ‘subsequent’ BP^1^**  **n/N, % [95% CI]** | **Clinical Hypertension with ‘total’ BP^1^**  **n/N, % [95% CI]** | **P-Value**  **(‘initial’ vs. ‘subsequent’ BP)^4^** | **P-Value**  **(‘initial’ vs ‘total’ BP)^4^** | **P-Value**  **(‘subsequent’ vs. ‘total’ BP)^4^** |
| --- | --- | --- | --- | --- | --- | --- |
| **Total Study Population** |  |  |  |  |  |  |
| Overall  (N=5,642) | 350/5642 6.2% [5.6%–6.9%] | 289/5642 5.1% [4.6%–5.7%] | 251/5642 4.4% [3.9%–5.0%] | **<0.001** | **<0.001** | **<0.001** |
| MP group (N=3,357) | 203/3357 6.0% [5.3%–6.9%] | 154/3357 4.6% [3.9%–5.3%] | 143/3357 4.3% [3.6%–5.0%] | **<0.001** | **<0.001** | **0.048** |
| AP group (N=2,285) | 147/2285 6.4% [5.5%–7.5%] | 135/2285 5.9% [5.0%–7.0%] | 108/2285 4.7% [3.9%–5.7%] | 0.300 | **<0.001** | **<0.001** |
| **Age 8-12 years^2^** |  |  |  |  |  |  |
| Overall (N=2,982) | 204/2982 6.8% [6.0%–7.8%] | 182/2982 6.1% [5.3%–7.0%] | 156/2982  5.2% [4.5%–6.1%] | 0.067 | **<0.001** | **<0.001** |
| MP group (N=1,770) | 126/1770 7.1% [6.0%–8.4%] | 106/1770 6.0% [5.0%–7.2%] | 96/1770 5.4% [4.5%–6.6%] | **0.012** | **<0.001** | **0.004** |
| AP group (N=1,212) | 78/1212 6.4% [5.2%–8.0%] | 76/1212 6.3% [5.0%–7.8%] | 60/1212 5.0% [3.9%–6.3%] | 0.823 | **0.009** | **0.006** |
| **Age 13-17 years** |  |  |  |  |  |  |
| Overall (N=2,660) | 146/2660 5.5% [4.7%–6.4%] | 107/2660 4.0% [3.3%–4.8%] | 95/2660 3.6% [2.9%–4.3%] | **<0.001** | **<0.001** | 0.052 |
| MP group (N=1,587) | 77/1587 4.9% [3.9%–6.0%] | 48/1587 3.0% [2.3%–4.0%] | 47/1587 3.0% [2.2%–3.9%] | **<0.001** | **<0.001** | 0.819 |
| AP group (N=1,073) | 69/1073 6.4% [5.1%–8.1%] | 59/1073 5.5% [4.3%–7.0%] | 48/1073 4.5% [3.4%–5.9%] | 0.174 | **<0.001** | **0.012** |
| **Males** |  |  |  |  |  |  |
| Overall (N=2,822) | 209/2822 7.4% [6.5%–8.4%] | 180/2822 6.4% [5.5%–7.3%] | 154/2822 5.5% [4.7%–6.4%] | **0.024** | **<0.001** | **<0.001** |
| MP group (N=1,684) | 122/1684 7.2% [6.1%–8.6%] | 93/1684 5.5% [4.5%–6.7%] | 85/1684 5.0% [4.1%–6.2%] | **0.002** | **<0.001** | 0.088 |
| AP group (N=1,138) | 87/1138 7.6% [6.2%–9.3%] | 87/1138 7.6% [6.2%–9.3%] | 69/1138 6.1% [4.8%–7.6%] | 1.000 | **0.013** | **0.002** |
| **Females** |  |  |  |  |  |  |
| Overall (N=2,820) | 141/2820 5.0% [4.3%–5.9%] | 109/2820 3.9% [3.2%–4.6%] | 97/2820 3.4% [2.8%–4.2%] | **0.001** | **<0.001** | **0.029** |
| MP group (N=1,673) | 81/1673 4.8% [3.9%–6.0%] | 61/1673 3.6% [2.8%–4.7%] | 58/1673 3.5% [2.7%–4.5%] | **0.002** | **<0.001** | 0.317 |
| AP group (N=1,147) | 60/1147 5.2% [4.1%–6.7%] | 48/1147 4.2% [3.2%–5.5%] | 39/1147 3.4% [2.5%–4.6%] | 0.096 | **<0.001** | 0.050 |
| **Non-Hispanic White** |  |  |  |  |  |  |
| Overall (N=1,645) | 85/1645 5.2% [4.2%–6.3%] | 75/1645 4.6% [3.7%–5.7%] | 63/1645 3.8% [3.0%–4.9%] | 0.251 | **0.003** | **0.007** |
| MP group (N=942) | 45/942 4.8% [3.6%–6.3%] | 36/942 3.8% [2.8%–5.2%] | 30/942 3.2% [2.2%–4.5%] | 0.128 | **0.004** | **0.034** |
| AP group (N=703) | 40/703 5.7% [4.2%–7.7%] | 39/703 5.5% [4.1%–7.5%] | 33/703 4.7% [3.4%–6.5%] | 0.876 | 0.194 | 0.083 |

|  | **Clinical Hypertension with ‘initial’ BP^1^**  **n/N, % [95% CI]** | **Clinical Hypertension with ‘subsequent’ BP^1^**  **n/N, % [95% CI]** | **Clinical Hypertension with ‘total’ BP^1^**  **n/N, % [95% CI]** | **P-Value**  **(‘initial’ vs. ‘subsequent’ BP)^4^** | **P-Value**  **(‘initial’ vs ‘total’ BP)^4^** | **P-Value**  **(‘subsequent’ vs. ‘total’ BP)^4^** |
| --- | --- | --- | --- | --- | --- | --- |
| **Non-Hispanic Black** |  |  |  |  |  |  |
| Overall (N=1,370) | 105/1370 7.7% [6.4%–9.2%] | 78/1370 5.7% [4.6%–7.0%] | 68/1370  5.0% [3.9%–6.2%] | **0.001** | **<0.001** | **0.033** |
| MP group (N=766) | 54/766 7.0% [5.4%–9.1%] | 33/766 4.3% [3.1%–6.0%] | 30/766 3.9% [2.8%–5.5%] | **0.001** | **<0.001** | 0.257 |
| AP group (N=604) | 51/604 8.4% [6.5%–10.9%] | 45/604 7.5% [5.6%–9.8%] | 38/604 6.3% [4.6%–8.5%] | 0.304 | **0.003** | 0.071 |
| **Hispanic** |  |  |  |  |  |  |
| Overall (N=1,697) | 107/1697 6.3% [5.2%–7.6%] | 92/1697 5.4% [4.4%–6.6%] | 80/1697 4.7% [3.8%–5.8%] | 0.087 | **<0.001** | **0.029** |
| MP group (N=1,130) | 80/1130 7.1% [5.7%–8.7%] | 64/1130 5.7% [4.5%–7.2%] | 62/1130 5.5% [4.3%–7.0%] | **0.011** | **0.001** | 0.564 |
| AP group (N=567) | 27/567 4.8% [3.3%–6.8%] \| | 28/567 4.9% [3.4%–7.0%] | 18/567 3.2% [2.0%–5.0%] | 0.869 | **0.039** | **0.018** |
| **Miscellaneous Ethnicity and Race** |  |  |  |  |  |  |
| Overall (N=930) | 53/930 5.7% [4.4%–7.4%] | 44/930 4.7% [3.5%–6.3%] | 40/930 4.3% [3.2%–5.8%] | 0.128 | **0.009** | 0.248 |
| MP group (N=519) | 24/519 4.6% [3.1%–6.8%] | 21/519 4.0% [2.7%–6.1%] | 21/519 4.0% [2.7%–6.1%] | 0.405 | 0.317 | 1.000 |
| AP group (N=411) | 29/411 7.1% [5.0%–9.9%] | 23/411 5.6% [3.8%–8.3%] | 19/411 4.6% [3.0%–7.1%] | 0.201 | **0.012** | 0.157 |
| **Underweight/healthy weight BMI^3^** |  |  |  |  |  |  |
| Overall (N=3,344) | 143/3344 4.3% [3.6%–5.0%] \| | 107/3344 3.2% [2.7%–3.9%] | 94/3344 2.8% [2.3%–3.4%] | **0.001** | **<0.001** | **0.033** |
| MP group (N=2,021) | 74/2021 3.7% [2.9%–4.6%] | 54/2021 2.7% [2.1%–3.5%] | 48/2021 2.4% [1.8%–3.1%] | **0.005** | **<0.001** | 0.083 |
| AP group (N=1,323) | 69/1323 5.2% [4.1%–6.5%] | 53/1323 4.0% [3.1%–5.2%] | 46/1323 3.5% [2.6%–4.6%] | **0.046** | **<0.001** | 0.162 |
| **Overweight/Obesity BMI^3^** |  |  |  |  |  |  |
| Overall (N=2,282) | 206/2282 9.0% [7.9%–10.3%] | 181/2282 7.9% [6.9%–9.1%] | 156/2282 6.8% [5.9%–7.9%] | **0.038** | **<0.001** | **<0.001** |
| MP group (N=1,325) | 128/1325 9.7% [8.2%–11.4%] | 99/1325 7.5% [6.2%–9.0%] | 94/1325 7.1% [5.8%–8.6%] | **0.001** | **<0.001** | 0.251 |
| AP group (N=957) | 78/957 8.2% [6.6%–10.1%] | 82/957 8.6% [7.0%–10.5%] | 62/957 6.5% [5.1%–8.2%] | 0.633 | **0.016** | **<0.001** |

Bold font indicates statistically significant results at p <0.05.

95% confidence intervals were calculated using the Wilson method for binomial proportions.

^1^Clinical hypertension refers to SBP and/or DBP in the hypertension range.

^2^N = 14 subjects aged <13 years had no height measurements and BP percentiles could not be calculated.

^3^N = 30 subjects had missing weight and/or height measurements; therefore, BMI could not be determined.

^4^P-values were calculated using McNemar’s tests and are unadjusted for multiple comparisons.

**Table S3a: Subjects with ‘initial’ BP in hypertension range: impact of repeat BP readings on BP category**

|  | **Overall** | **MP group** | **AP group** | **P-Value^2^** |
| --- | --- | --- | --- | --- |
| **Subjects with ‘hypertension’ range SBP_1_** | **^1^N=237** | **N=146** | **N=91** |  |
| SBP_2+3_ Normal | 28 (11.8%) | 7 (4.8%) | 21 (23.1%) | **<0.001** |
| SBP_2+3_ Elevated | 63 (26.6%) | 37 (25.3%) | 26 (28.6%) |  |
| SBP_2+3_ Hypertension | 146 (61.6%) | 102 (69.9%) | 44 (48.4%) |  |
|  |  |  |  |  |
| SBP_1+2+3_ Normal | 14 (5.9%) | 3 (2.1%) | 11 (12.1%) | **0.003** |
| SBP_1+2+3_ Elevated | 63 (26.6%) | 36 (24.7%) | 27 (29.7%) |  |
| SBP_1+2+3_ Hypertension | 160 (67.5%) | 107 (73.3%) | 53 (58.2%) |  |
|  |  |  |  |  |
| **Subjects with ‘hypertension’ range DBP_1_** | **^1^N=153** | **N=70** | **N=83** |  |
| DBP_2+3_ Normal | 85 (55.6%) | 44 (62.9%) | 41 (49.4%) | 0.25 |
| DBP_2+3_ Elevated | 10 (6.5%) | 4 (5.7%) | 6 (7.2%) |  |
| DBP_2+3_ Hypertension | 58 (37.9%) | 22 (31.4%) | 36 (43.4%) |  |
|  |  |  |  |  |
| DBP_1+2+3_ Normal | 72 (47.1%) | 37 (52.9%) | 35 (42.2%) | 0.18 |
| DBP_1+2+3_ Elevated | 10 (6.5%) | 6 (8.6%) | 4 (4.8%) |  |
| DBP_1+2+3_ Hypertension | 71 (46.4%) | 27 (38.6%) | 44 (53.0%) |  |
| ^1^N = 14 subjects aged <13 years had no height measurements and BP percentiles could not be calculated.  ^2^P-values compare MP and AP groups and were calculated using chi-squared or Fisher’s exact tests, as appropriate.  ^3^SBP1/DBP1 = initial SBP/DBP reading; SBP2+3/DBP2+3 = subsequent SBP/DBP; SBP1+2+3/DBP1+2+3 = total SBP/DBP. | | | | |

**Table S3b**: **Subjects with ‘initial’ BP in ‘elevated BP’ range: impact of repeat BP readings on BP category**

|  | **Overall** | **MP group** | **AP group** | **P-Value^2^** |
| --- | --- | --- | --- | --- |
| **Subjects with ‘elevated BP’ range SBP_1_** | **^1^N=501** | **N=330** | **N=171** |  |
| SBP_2+3_ Normal | 220 (43.9%) | 156 (47.3%) | 64 (37.4%) | **0.04** |
| SBP_2+3_ Elevated | 242 (48.3%) | 154 (46.7%) | 88 (51.5%) |  |
| SBP_2+3_ Hypertension | 39 (7.8%) | 20 (6.1%) | 19 (11.1%) |  |
|  |  |  |  |  |
| SBP_1+2+3_ Normal | 186 (37.1%) | 132 (40.0%) | 54 (31.6%) | **0.02** |
| SBP_1+2+3_ Elevated | 290 (57.9%) | 187 (56.7%) | 103 (60.2%) |  |
| SBP_1+2+3_ Hypertension | 25 (5.0%) | 11 (3.3%) | 14 (8.2%) |  |
|  |  |  |  |  |
| **Subjects with ‘elevated BP’ range DBP_1_** | **N=66** | **N=40** | **N=26** |  |
| DBP_2+3_ Normal | 46 (69.7%) | 25 (62.5%) | 21 (80.8%) | 0.09 |
| DBP_2+3_ Elevated | 14 (21.2%) | 12 (30.0%) | 2 (7.7%) |  |
| DBP_2+3_ Hypertension | 6 (9.1%) | 3 (7.5%) | 3 (11.5%) |  |
|  |  |  |  |  |
| DBP_1+2+3_ Normal | 45 (68.2%) | 25 (62.5%) | 20 (76.9%) | 0.46 |
| DBP_1+2+3_ Elevated | 18 (27.3%) | 13 (32.5%) | 5 (19.2%) |  |
| DBP_1+2+3_ Hypertension | 3 (4.6%) | 2 (5.0%) | 1 (3.9%) |  |
| ^1^N = 14 subjects aged <13 years had no height measurements and BP percentiles could not be calculated.  ^2^P-values compare MP and AP groups and were calculated using chi-squared or Fisher’s exact tests, as appropriate.  ^3^SBP1/DBP1 = initial SBP/DBP reading; SBP2+3/DBP2+3 = subsequent SBP/DBP; SBP1+2+3/DBP1+2+3 = total SBP/DBP. | | | | |

**Table S3c**: **Subjects with ‘initial’ BP in normal range: impact of repeat BP readings on BP category**

|  | **Overall** | **MP group** | **AP group** | **P-value^2^** |
| --- | --- | --- | --- | --- |
| **Subjects with normal SBP_1_** | **^1^N=4,904** | **N=2,881** | **N=2,023** |  |
| SBP_2+3_ Normal | 4,762 (97.1%) | 2,816 (97.7%) | 1,946 (96.2%) | **0.002** |
| SBP_2+3_ Elevated | 128 (2.6%) | 61 (2.1%) | 67 (3.3%) |  |
| SBP_2+3_ Hypertension | 14 (0.3%) | 4 (0.1%) | 10 (0.5%) |  |
|  |  |  |  |  |
| SBP_1+2+3_ Normal | 4,837 (98.6%) | 2,858 (99.2%) | 1,979 (97.8%) | **<0.001** |
| SBP_1+2+3_ Elevated | 65 (1.3%) | 23 (0.8%) | 42 (2.1%) |  |
| SBP_1+2+3_ Hypertension | 2 (0.04%) | 0 (0%) | 2 (0.1%) |  |
|  |  |  |  |  |
| **Subjects with normal DBP_1_** | **^1^N=5,423** | **N=3,247** | **N=2,176** |  |
| DBP_2+3_ Normal | 5,336 (98.4%) | 3,217 (99.1%) | 2,119 (97.4%) | **<0.001** |
| DBP_2+3_ Elevated | 25 (0.5%) | 15 (0.5%) | 10 (0.5%) |  |
| DBP_2+3_ Hypertension | 62 (1.1%) | 15 (0.5%) | 47 (2.2%) |  |
|  |  |  |  |  |
| DBP_1+2+3_ Normal | 5,386 (99.3%) | 3,236 (99.7%) | 2,150 (98.8%) | **0.001** |
| DBP_1+2+3_ Elevated | 17 (0.3%) | 5 (0.2%) | 12 (0.6%) |  |
| DBP_1+2+3_ Hypertension | 20 (0.4%) | 6 (0.2%) | 14 (0.6%) |  |
| ^1^N = 14 subjects aged <13 years had no height measurements and BP percentiles could not be calculated.  ^2^P-values compare MP and AP groups and were calculated using chi-squared or Fisher’s exact tests, as appropriate.  ^3^SBP1/DBP1 = initial SBP/DBP reading; SBP2+3/DBP2+3 = subsequent SBP/DBP; SBP1+2+3/DBP1+2+3 = total SBP/DBP. | | | | |

**Table S3d: Subjects with ‘initial’ BP in ‘EBP’ or normal range: impact of repeat BP readings on BP category**

|  | **Overall** | **MP group** | **AP group** | **P-Value^2^** | |
| --- | --- | --- | --- | --- | --- |
| **Subjects with ‘EBP’ range or normal SBP_1_** | ^1^N=5,405 | N=3,211 | N=2,194 |  | |
| SBP_2+3_ Normal | 4,982 (92.2%) | 2,972 (92.6%) | 2,010 (91.6%) | 0.092 | |
| SBP_2+3_ Elevated | 370 (6.9%) | 215 (6.7%) | 155 (7.1%) |  | |
| SBP_2+3_ Hypertension | 53 (1.0%) | 24 (0.8%) | 29 (1.3%) |  | |
|  |  |  |  |  | |
| SBP_1+2+3_ Normal | 5,023 (92.9%) | 2,990 (93.1%) | 2,033 (92.7%) | 0.139 | |
| SBP_1+2+3_ Elevated | 355 (6.6%) | 210 (6.5%) | 145 (6.6%) |  | |
| SBP_1+2+3_ Hypertension | 27 (0.5%) | 11 (0.3%) | 16 (0.7%) |  | |
|  |  |  |  |  | |
| **Subjects with ‘EBP’ range or normal DBP_1_** | N=5,489 | N=3,287 | N=2,202 |  | |
| DBP_2+3_ Normal | 5,382 (98.1%) | 3,242 (98.6%) | 2,140 (97.2%) | <0.001 | |
| DBP_2+3_ Elevated | 39 (0.7%) | 27 (0.8%) | 12 (0.6%) |  | |
| DBP_2+3_ Hypertension | 68 (1.2%) | 18 (0.6%) | 50 (2.3%) |  | |
|  |  |  |  |  | |
| DBP_1+2+3_ Normal | 5,431 (98.9%) | 3,261 (99.2%) | 2,170 (98.6%) | 0.028 | |
| DBP_1+2+3_ Elevated | 35 (0.6%) | 18 (0.6%) | 17 (0.8%) |  | |
| DBP_1+2+3_ Hypertension | 23 (0.4%) | 8 (0.2%) | 15 (0.7%) |  | |
| ^1^N = 14 subjects aged <13 years had no height measurements and BP percentiles could not be calculated.  ^2^P-values compare MP and AP groups and were calculated using chi-squared or Fisher’s exact tests, as appropriate.  ^3^SBP1/DBP1 = initial SBP/DBP reading; SBP2+3/DBP2+3 = subsequent SBP/DBP; SBP1+2+3/DBP1+2+3 = total SBP/DBP. | | | | |  |

**Table S4: Group level BP readings**

|  | **Overall**  **N=5,656**  **Mean (SD)**  **Median (IQR)** | **MP group**  **N=3,365**  **Mean (SD)**  **Median (IQR)** | **AP group**  **N=2,291**  **Mean (SD)**  **Median (IQR)** | **P-value^1^** |
| --- | --- | --- | --- | --- |
| **SBP1** | 105.4 (10.4)  105.0 (98.0, 112.0) | 105.7 (10.1)  106.0 (98.0, 112.0) | 104.8 (10.8)  104.0 (98.0, 111.0) | **0.001** |
| **SBP2** | 105.1 (10.3)  105.0 (98.0, 112.0) | 105.5 (10.0)  106.0 (98.0, 112.0) | 104.6 (10.6)  104.0 (97.0, 111.0) | **0.001** |
| **SBP3** | 105.1 (10.4)  104.0 (98.0, 112.0) | 105.5 (10.2)  106.0 (98.0, 112.0) | 104.5 (10.7)  104.0 (97.0, 111.0) | **<0.001** |
| **Mean SBP_2+3_** | 105.1 (10.0)  105.0 (98.0, 111.5) | 105.5 (9.9)  105.0 (98.0, 112.0) | 104.6 (10.2)  104.0 (97.5, 111.0) | **<0.001** |
| **Mean SBP_1+2+3_** | 105.2 (9.9)  104.7 (98.3, 111.3) | 105.6 (9.7)  105.3 (98.7, 112.0) | 104.6 (10.0)  104.0 (98.0, 111.0) | **<0.001** |
| **DBP1** | 59.3 (10.6)  60.0 (53.0, 66.0) | 57.2 (11.3)  58.0 (50.0, 66.0) | 62.4 (8.6)  62.0 (57.0, 67.0) | **<0.001** |
| **DBP2** | 59.0 (10.6)  60.0 (52.0, 66.0) | 57.0 (11.1)  58.0 (50.0, 66.0) | 62.0 (9.1)  61.0 (56.0, 67.0) | **<0.001** |
| **DBP3** | 59.1 (10.7)  60.0 (52.0, 66.0) | 57.2 (11.4)  58.0 (50.0, 66.0) | 61.9 (9.0)  61.0 (56.0, 67.0) | **<0.001** |
| **Mean DBP_2+3_** | 59.1 (10.1)  59.5 (53.0, 66.0) | 57.1 (10.7)  57.0 (50.0, 65.0) | 61.9 (8.3)  61.5 (56.0, 67.0) | **<0.001** |
| **Mean DBP_1+2+3_** | 59.1 (9.8)  59.3 (53.3, 65.7) | 57.2 (10.4)  57.3 (50.0, 64.7) | 62.1 (7.9)  61.7 (56.7, 67.0) | **<0.001** |

^1^P-values compare MP and AP groups and were calculated using Wilcoxon rank-sum tests.

**Table S5: Descriptive patterns of three within-visit systolic and diastolic BP readings in the manual and automated groups**

| **Pattern** | **Overall (N=5,656)** | **MP group (N=3,365)** | **AP group (N=2,291)** |
| --- | --- | --- | --- |
| **Highest SBP was first reading** | 2,499 (44.2%) | 1,548 (46.0%) | 951 (41.5%) |
| **Highest SBP was second reading** | 1,722 (30.5%) | 1,012 (30.1%) | 710 (31.0%) |
| **Highest SBP was third reading** | 1,435 (25.4%) | 805 (23.9%) | 630 (27.5%) |
| **Highest DBP was first reading** | 2,400 (42.4%) | 1,383 (41.1%) | 1,017 (44.4%) |
| **Highest DBP was second reading** | 1,687 (29.8%) | 1,023 (30.4%) | 664 (29.0%) |
| **Highest DBP was third reading** | 1,569 (27.7%) | 959 (28.5%) | 610 (26.6%) |
| **SBP decreased throughout** | 692 (12.2%) | 357 (10.6%) | 335 (14.6%) |
| **DBP decreased throughout** | 803 (14.2%) | 434 (12.9%) | 369 (16.1%) |
| **SBP increased throughout** | 582 (10.3%) | 300 (8.9%) | 282 (12.3%) |
| **DBP increased throughout** | 676 (12.0%) | 425 (12.6%) | 251 (11.0%) |

Reprinted from “Within-visit blood pressure variability in children and adolescents in the National Health and Nutrition Examination Survey (2013-2020),” by Riar SK, Gillespie S, South AM, J Hypertens. 2025 Jul 1;43(7):1158-1168. Table 3. Copyright 2025 by Wolters Kluwer Health, Inc. Used with permission.

**Figures:**

**Figure S1a: ‘Clinical’ hypertension stratified by age, sex, and BMI (MP vs. AP groups)**

**Figure S1b: ‘Clinical’ hypertension stratified by race-ethnicity (MP vs. AP groups)**

**Figure S2a: Subjects with ‘initial’ SBP in hypertension range: impact of ‘subsequent’ vs. ‘total’ BP on BP classification**

Each column includes subjects with ‘initial’ SBP in hypertension range in MP or AP group as indicated. Impact of ‘subsequent’ SBP on BP category (normal, EBP or hypertension) is shown in the first two columns. The next two columns show change in BP category with ‘total’ SBP.

**Figure S2b: Subjects with ‘initial’ DBP in hypertension range: impact of ‘subsequent’ vs. ‘total’ BP on BP classification**

Each column includes subjects with ‘initial’ DBP in hypertension range in MP or AP group as indicated. Impact of ‘subsequent’ DBP on BP category (normal, EBP or hypertension) is shown in the first two columns. The next two columns show change in BP category with ‘total’ DBP.

**Figure S2c: Subjects with ‘initial’ SBP in EBP range: impact of ‘subsequent’ vs. ‘total’ BP on BP classification**

Each column includes subjects with ‘initial’ SBP in EBP range in MP or AP group as indicated. Impact of ‘subsequent’ SBP on BP category (normal, EBP or hypertension) is shown in the first two columns. The next two columns show change in BP category with ‘total’ SBP.
